# The Role of Host Genetics and Intestinal Microbiota and Metabolome as a New Insight into IBD Pathogenesis

**DOI:** 10.3390/ijms25179589

**Published:** 2024-09-04

**Authors:** Oliwia Zakerska-Banaszak, Joanna Zuraszek-Szymanska, Piotr Eder, Karolina Ladziak, Ryszard Slomski, Marzena Skrzypczak-Zielinska

**Affiliations:** 1 Institute of Human Genetics, Polish Academy of Sciences, 60-479 Poznan, Polandslomski@up.poznan.pl (R.S.); 2 Department of Gastroenterology, Dietetics and Internal Medicine, Poznan University of Medical Sciences, 60-355 Poznan, Poland; piotreder@ump.edu.pl

**Keywords:** ulcerative colitis, Crohn’s disease, gut microbiota, short-chain fatty acids, host genetics, metabolome

## Abstract

Inflammatory bowel disease (IBD) is an incurable, chronic disorder of the gastrointestinal tract whose incidence increases every year. Scientific research constantly delivers new information about the disease and its multivariate, complex etiology. Nevertheless, full discovery and understanding of the complete mechanism of IBD pathogenesis still pose a significant challenge to today’s science. Recent studies have unanimously confirmed the association of gut microbial dysbiosis with IBD and its contribution to the regulation of the inflammatory process. It transpires that the altered composition of pathogenic and commensal bacteria is not only characteristic of disturbed intestinal homeostasis in IBD, but also of viruses, parasites, and fungi, which are active in the intestine. The crucial function of the microbial metabolome in the human body is altered, which causes a wide range of effects on the host, thus providing a basis for the disease. On the other hand, human genomic and functional research has revealed more loci that play an essential role in gut homeostasis regulation, the immune response, and intestinal epithelial function. This review aims to organize and summarize the currently available knowledge concerning the role and interaction of crucial factors associated with IBD pathogenesis, notably, host genetic composition, intestinal microbiota and metabolome, and immune regulation.

## 1. Introduction

Inflammatory bowel disease (IBD), which includes ulcerative colitis (UC) and Crohn’s disease (CD), affects around 7 million people worldwide. As an inflammatory, chronic, and still incurable disease that permanently reduces the quality of patient’s lives, it currently poses a serious challenge to the medical sector and human society [1,2]. Genetic, immunological, and environmental factors have been involved in IBD pathogenesis. Nevertheless, despite the technological advancements in molecular biology, the etiology of IBD is still not fully understood [3]. Recent studies demonstrated that besides the host genetic predispositions, compositional changes in the gut bacterial microbiota are associated with IBD [4]. The fundamental roles of the gut microbial community in terms of host health include maintaining intestinal homeostasis, driving the immune response, and maintaining the function of the epithelial barrier. A growing body of evidence defining the host–microbe relationships, including bacteria, viruses, fungi, parasites, and their specific metabolites, is crucial to understanding IBD pathogenesis. Focusing on new insights that pertain to the relationship of microbiota and metabolome to host genetics, we want to systematize, summarize, and discuss the data on IBD pathogenesis.

## 2. Gut Microbiota and IBD

The human gut microbiota constitutes an ecosystem composed of over 100 trillion various microorganisms, mainly bacteria, including those commonly found in the healthy intestine species like *Bacteroides fragilis*, *Faecalibacterium prausnitzii*, *Lactobacillus rhamnosus*, *Bifidobacterium longum*, *Clostridium leptum*, and *Ruminococcus bromii*, but also viruses, fungi, archaea, and parasites. Physiologically, the composition of microbes in the intestine is highly diverse among healthy individuals. However, they are symbiotic with the host, forming gut-associated lymphoid tissue (GALT) and producing vitamins and metabolites necessary for homeostasis and human well-being. The human intestine constitutes a nutrient-rich environment for the microbial community. This interaction is regulated by environmental factors and host genetics [5]. In IBD patients, a change in the balance between commensal and pathogenic microorganisms in the gut (dysbiosis) is observed also in the metabolic profile and, finally, in the microbiota interaction with the host [4,6]. Then, the abundance of bacteria that coexist with the host without causing disease, like those from genera *Bifidobacterium* and *Lactobacillus*, decreases in favor of species that can worsen inflammation and intestinal damage in a state of dysbiosis, such as adherent-invasive *Escherichia coli* and *Mycobacterium avium* subsp. *paratuberculosis*. This microbial imbalance is vital because metabolites, including, among others, short-chain fatty acids (SCFAs), and lipopolysaccharides (LPS) produced by specific taxa can influence the expression of anti- and pro-inflammatory cytokines, T helper (Th) 1, 2, and 17, and regulatory T cells (T-reg) [7,8,9]. Studies have revealed that in germ-free mice, a reduced amount of Th 17 cells, lymphocytes, and immunoglobulin (Ig) A in the intestinal mucosa was observed compared to the controls [10,11,12]. Animal studies confirmed the role of microbiota in the development of inflammatory diseases. They revealed that dysbiotic bacteria transferred to healthy mice induce inflammation. Furthermore, it was proved that gut microbiota obtained from IBD patients alter the immune response and exacerbate UC in mice [13]. At the same time, naive CD4+ lymphocytes transferred from healthy mice to those that lack T and B lymphocytes may induce UC, but the susceptibility depends on the gut microbial composition [14].

### 2.1. Bacterial Dysbiosis

Alterations in the bacterial composition in the intestine of IBD patients have already been well described. In general, dysbiosis in IBD is observed as a state of a reduced abundance of commensal taxa with a concomitant increase in pathogenic bacteria. Moreover, the intestinal bacterial community shows decreased diversity and is less stable in those patients than healthy controls [15]. In a healthy population, the most represented bacterial phyla are *Firmicutes*, *Bacteroidetes*, *Proteobacteria*, *Actinobacteria*, and *Verrucomicrobia* [16].

Global studies indicate that the primary shifts at the phylum level include reducing *Firmicutes* and *Bacteroidetes* in both CD and UC cases [4,17]. Moreover, the ratio of *Firmicutes/Bacteroidetes* is lower, which is a principal indicator of bacterial balance in the gut. Many beneficial taxa are reduced from the *Firmicutes* phylum, such as *Faecalibacterium prausnitzii*, *Clostridium XIVa*, *IV*, *Lactobacillus*, and *Roseburia faecis*, producing butyrate and SCFA, which are of great importance in the host [18]. *F. prausnitzii*, known as a biomarker of a healthy gut, plays a key role because of its anti-inflammatory metabolite, which inhibits the NF-kB pathway in epithelial cell lines [19]. *Verrucomicrobia* is also mostly reported to be lowered in IBD patients. The commensal *Akkermansia* (*A.*) *muciniphila* belongs to this bacterial phylum and has a pivotal function in the intestine due to ameliorating mucosal inflammation either via microbe–host interactions, which protect the gut barrier function and reduce the levels of inflammatory cytokines, or by improving the microbial community. This probiotic potential of *A. muciniphila* could make this bacterium an important agent in UC treatment. A deficiency of *A. muciniphila* in UC patients could result from the decreased level of mucins in the gut mucosa, which are the source of energy for these bacteria. Furthermore, *A. muciniphila* supports the intestinal barrier via its protein Amuc_1100 [20].

On the other hand, in IBD subjects, a marked increase in aerobes is observed. This mainly concerns *Proteobacteria* and *Actinobacteria* phyla bacteria, including *E. coli* and *Desulfovibrio*, which have a pro-inflammatory influence on the intestinal mucosa [21]. This tendency is associated with inflammation, an oxidative process that may induce an overgrowth of bacteria adherent to the colonic mucosa with a higher tolerance to oxygen [21]. Among IBD patients, the enrichment of mucolytic bacteria is noted with, e.g., *Ruminococcus gnavus*. This process weakens the intestine’s defensive abilities against pathogenic bacteria.

In IBD (both CD and UC), general changes in gut microbial composition are common. However, in CD, the intestinal microbiota is more altered and unstable than in UC. Moreover, specific shifts are reported, depending on disease location and activity [15].

### 2.2. Virome Dysbiosis

Viruses infecting prokaryotes and eukaryotes constitute a part of the gut microbial community. They are involved in host ecosystem dynamics and provide antibiotic-resistance genes. The human intestinal virome is stable and personalized and mainly consists of bacteriophages [22]. Certain stress factors can induce the lytic cycle and viral replication. In IBD patients, virome composition changes result from bacterial alterations in the intestine [23]. Several independent studies detected the *Caudovirales* phage sequence in both CD and UC samples of stool, mucosal washes, or biopsies [24]. Among *Caudovirales* phage families, *Siphoviridae*, *Myoviridae*, and *Podoviridae* were present. An increased abundance of the *Caudovirales* phage in the intestine correlated with decreased bacterial diversity and mucosal inflammation [25]. Furthermore, Ungaro et al. revealed an increased presence of *Herpesviridae* in the mucosa of CD patients and *Hepadnaviridae* in UC patients [26]. Recent investigations conducted on germ-free mice demonstrated that *Lactobacillus*, *Bacteroides*, and *Escherichia* infecting phages and phage DNA enhance the inflammatory process in the gut. There is also evidence that phages infecting eukaryotic cells play a role in IBD pathogenesis due to integration with the host genome and affecting intestinal cells [27].

### 2.3. Mycobiome Dysbiosis

Fungi’s contribution to IBD pathogenesis has been suspected since their pro-inflammatory influence was initially discovered [28]. Mycobiome dysbiosis has been reported extensively in IBD patients. Fungi constitute approximately 0.1% of human intestine microbiota and are mainly composed of the *Ascomycota* and *Basidiomycota* phyla, where the most common genera are *Saccharomyces*, *Debaryomyces*, *Penicillium*, *Kluyveromyces*, and *Candida* [29].

Mycobiome dysbiosis is characterized by an increased ratio of *Basidiomycota* to *Ascomycota*. Higher gut mycobiota diversity was shown in individuals with ileal CD, while decreased diversity was present in cases of UC and colonic CD [29]. Studies proved that an abundance of protective *Saccharomyces cerevisiae* was detected in IBD subjects at lower levels compared to the controls, in contrast to *Candida albicans*, *Candida tropicalis*, *Clavispora lusitaniae*, *Cyberlindnera jadinii*, and *Kluyveromyces marxianus*, which were elevated [30]. A different influence of fungi on the inflammation process was discovered. *Candida albicans* can positively modulate susceptibility to inflammation, while *Saccharomyces boulardii* or *Saccharomyces cerevisiae* has the opposite effect. Knowledge about the interactions between intestinal mycobiota and the human immune system comes mainly from research on *Candida* and *Saccharomyces*; however, how the immune system recognizes fungal invasion or colonization in the gut lumen has not been fully discovered. In particular, the *Candida* infection significantly raised the plasma IL1β and TNFα levels in the experimental model of colitis [31]. Furthermore, *Candida albicans* has been observed to expand in active IBD patients compared to IBD in remission [29]. Meanwhile, a higher abundance of *Aspergillus*, *Wickerhamomyces*, *Candida*, and *Sterigmatomyces*, and a lower richness of *Alternaria, Penicillium*, *Exophiala*, *Emericella*, *Acremonium*, *Epicoccum*, and *Trametes*, was shown by Qiu et al. in UC patients compared to healthy subjects [32].

The evidence of the direct contribution of fungi to IBD includes several mechanisms of action. *Candida albicans* has been proven to be one of the most common IBD-associated fungal species because of its virulence factors, such as hyphal formation, toxin secretion, adherence ability, and resistant biofilm formation. By secretion of candida lysin, a toxic protein, this fungal species induces damage to the host’s epithelial membrane and intestinal macrophages. *Candida albicans* can convert from commensal fungi to hyphal structures, but a properly functioning host immune system maintains its nonpathogenic form. Furthermore, it was proved that the interaction of *Candida tropicalis* with *Serratia marcescens* and *E. coli* results in increased biofilm production [33,34]. 

Different studies indicate a complex relationship between the bacteria and fungi in the gut microbiota; specific alterations are present in IBD. In a study on fungal microbiota dysbiosis in IBD performed by Sokol et al., a positive correlation was found between the high abundance of protective *Saccharomyces* and several bacteria such as *Bifidobacterium*, *Blautia*, *Roseburia*, and *Ruminococcus* in IBD cases [29]. 

Interestingly, there is growing evidence concerning an association between specific fungal abundance and the genotype of host genes. Such an association was found, for example, between the *CLEC7A* gene, coding for dectin-1 and an abundance of *Malassezia sympodialis*, *Aspergillus*, and *Candida* in UC; between the *CARD9* gene and an abundance of *Saccharomyces cerevisiae*; and between the *TLR1* gene (coding for toll-like receptor 1) and *Malassezia sympodialis* in IBD [35]. Dectin-1, a transmembrane protein expressed by host macrophages and monocytes, interacts with the β-glucans located in the layer of the fungal cell wall. These proteins are important for host myeloid cell recognition, inflammatory cytokines release, and T-cell development. It was proved that the interaction between dectin-1 and β-glucans plays a crucial role in response to *Candia* species and many other fungi [36].

Recent observations supporting the fact that fungal pathogens disturb the host immune system indicate that in CD patients, increased antibodies against fungal targets are identified long before diagnosis [37].

### 2.4. Parasites

Intestinal parasites are well known for their pathogenic potential in the host organism. However, recent studies revealed the commensal role of some of these in the human gut, as in maintaining intestinal homeostasis. Research demonstrated the presence of over 15 different genera of protozoa (amoebozoans, flagellates, ciliates, stramenopiles, and apicomplexans) classified as parasites but belonging to the commensals of the GI tract [38,39]. The impact of protozoan parasites on the development and progression of IBD has been analyzed with a particular focus on *Blastocystis hominis*, which is associated with different gastrointestinal disturbances. Surprisingly, it was observed that the frequency of *Blastocystis hominis* infection was lower in IBD patients compared to healthy controls and in patients with active UC in contrast to individuals in remission [40]. Additionally, *Blastocystis hominis* were found to be associated with a higher abundance of *Clostridia*, *Ruminococcaceae*, and *Prevotellaceae* as well as butyrate-producing *Faecalibacterium* and *Roseburia*, but a lower abundance of *Enterobacteriaceae*. *Blastocystis hominis* colonization was not linked with colitis-specific dysbiosis but was recognized as a common element of healthy human microbiota acting protectively in gastrointestinal diseases [41]. On the other hand, some studies demonstrated the opposite results, where *Blastocystis hominis* was in a higher abundance in UC patients compared to the controls [42].

However, numerous protozoan parasites are pathogenic and may induce intestinal inflammation and complicate the course of the disease. Studies prove that *Entamoeba histolytica* might provoke colitis [43]. Moreover, it is known that *Cryptosporidium parvum* may exacerbate the course of IBD [44]. 

Finally, due to insufficient data in this area, the relationship between parasites and other intestinal microbiota in IBD pathogenesis is a major issue that requires further research and exploration. On the other hand, studies have shown the potential of human helminths in regulating the immune system in IBD. Helminth-derived product therapy is currently being studied in animal colitis models [45].

Microbial dysbiosis in IBD is well-established, and numerous review studies in this area are available [46]. Nevertheless, understanding these characteristic changes in the microbiota composition in patients with IBD, as mentioned in the conclusions of numerous independent world studies, is important to fully discovering the disease mechanism. It is known that some shifts in taxonomic levels are closely related to the development of intestinal inflammation. However, it is still unclear whether intestinal dysbiosis is the cause or the consequence of inflammation.

## 3. Metabolomic Changes of Intestinal Microbiota

Once the dysbiosis phenomenon had been described in IBD patients, studies on changes in the profile of intestinal metabolites began. Global investigations showed disturbances in the metabolic balance in patients’ stools, urine, blood, tissue, and breath [47]. Shifts in the metabolomic profile of IBD patients result from impairments in gut microbiota composition. In general, functionally essential and biologically active metabolites have been observed to be depleted in major IBD cases [48].

### 3.1. SCFAs

Particularly substantial and beneficial metabolites come in the form of SCFAs, products of microbial fermentation, including acetate, propionate, and butyrate, which have anti-inflammatory properties. Bacteria-producing SCFAs (such as *Bacteroidetes*, *Clostridium clusters IV* and *XIVa*, and *Faecalibacterium prausnitzii*) are reported to be decreased in IBD. SCFAs constitute a source of energy for colonic epithelial cells, modulate intestinal motility, induce the activity of T cells in the colon, and are also responsible for maintaining remission in UC individuals [49]. Acetate and propionate exert extra-intestinal roles, acting as metabolic substrates for lipogenesis and gluconeogenesis. Studies showed that butyrate leads to the downregulation of overexpressed inflammatory pathway genes in UC patients [50].

### 3.2. Bile Acid

Secondary bile acids are products of intestinal microbiota metabolism. They act as signaling factors on nuclear receptors, which control the communication process between intestinal microbiota and the host immune system [51]. Four decades of research were needed to establish IBD’s correlation with bile acid. Research data demonstrated that oral administration of secondary bile acids in mice alleviated the severity of colitis. Bile acid malabsorption was reported to be a common reason for diarrhea in CD and UC patients [52]. Microbial dysbiosis associated with IBD leads to bile acid dysmetabolism and may disturb the anti-inflammatory effect of bile acids [53].

### 3.3. Hydrogen Sulfide

Hydrogen sulfide is generated by gut microbiota in two biochemical pathways—by sulfate-reducing bacteria (SRB), such as *Desulfotomaculum*, *Desulfosporosinus*, *Thermodesulfobacterium*, *Thermodesulfovibrio*, and *Desulfovibrio*, or by bacteria that ferment sulfur-containing amino acids, such as *Fusobacterium nucleatum*, *Atopobium* spp., *Gemella sanguinis*, *Micromonas micros*, *Streptococcus* spp., *Actinomyces* spp., *Eubacterium* spp., *Veilonella* spp., *Prevotella* spp., *Campylobacter* spp., and *Selenomonas* spp. [54]. Studies showed enrichment of hydrogen sulfide producers in CD patients. Furthermore, it is also correlated with the disease activity. Individuals with active CD revealed an increase in *Enterococcus*, *Fusobacterium*, *Haemophilus*, *Megasphaera*, and *Campylobacter*, while *Roseburia*, *Christensenellaceae*, *Oscillibacter*, and *Odoribacter* were more abundant in subjects with inactive CD [55]. Hydrogen sulfide shows genotoxic and cytotoxic activity at higher concentrations by affecting the genes responsible for cell cycle progression, DNA repair, and inflammatory response. Hydrogen sulfide may also inhibit the cytochrome c oxidase activity of mitochondrial respiration [56]. Moreover, functional profiling using shotgun metagenomics confirmed the dysregulation of metabolic pathways involved in sulfur metabolism in CD and UC [55]. In IBD, higher hydrogen sulfide generation is confirmed, and disturbed intestinal detoxification of hydrogen sulfide is also proved. There is also evidence that hydrogen sulfide can block the use of butyrate by colonocytes [57].

### 3.4. Aromatic Amino Acids

Aromatic amino acids, including histidine, phenylalanine, tryptophan, and tyrosine, are essential in the metabolism and function of gut microbiota. These amino acids serve as precursors for various bioactive compounds, such as neurotransmitters and hormones, which play a significant role in gut–brain communication [58]. The most important one for the metabolomic changes is tryptophan, a precursor to serotonin, melatonin, nicotinamide, and vitamin B3. These metabolites are associated with various physiological functions, including intestinal inflammation, the epithelial barrier, and the host’s energy homeostasis. The metabolism of tryptophan occurs through three major pathways in the gastrointestinal tract: kynurenine (KYN), indole, and 5-hydroxytryptamine (5-HT). These pathways are crucial in intestinal inflammation regulation by influencing the balance of pro- and anti-inflammatory cytokines, modulating the functions of various immune cells, and impacting the intestinal microbiota composition [59]. The gut microbiota can metabolize it into a range of indole metabolites, some of which can act as ligands for the aryl hydrocarbon receptor (AhR), which has been implicated in IBD pathogenesis [60]. Dysbiosis leads to a loss of microbial activation of tryptophan, causing increased metabolism down the kynurenine pathway. In patients with Crohn’s disease, reduced expression of AhR in inflamed mucosal samples was reported. Recent studies demonstrated that the commensal bacteria *Peptostreptococcus russellii* could reduce susceptibility to colitis by metabolizing tryptophan to IA (an AhR agonist), improving cell differentiation and reducing inflammatory signals [61].

### 3.5. Succinate

An increased interest in succinate (tricarboxylic acid cycle intermediate) metabolism in the context of IBD is observed. In the human organism, succinate is a pro-inflammatory signaling molecule. Furthermore, studies proved it mediates the macrophage response to lipopolysaccharide [62]. Higher levels of succinate were found in the serum of Crohn’s disease patients and in the fecal samples of both CD and UC subjects [63]. However, a lower level of succinate was shown in the urine of IBD patients, compared to healthy controls. Studies also revealed a decreased abundance of intestinal *Phascolarctobacterium* in IBD, which is responsible for succinate metabolism [64].

It is already well-documented that these are only a few of the most important metabolites involved in the main metabolic pathways that impact inflammation. Nevertheless, it should be kept in mind that research in this area is still ongoing, and untargeted metabolomics and different small-molecule detection methods combined with functional metagenomics will provide knowledge about new bioactive molecules relevant to IBD in time [65].

### 3.6. Sphingolipids

Bacteria from the Bacteroidetes phylum, including genera such as Bacteroides, *Parabacteroides*, *Prevotella*, *Porphyromonas*, and *Flectobacillus*, are known to produce sphingolipids, which have a significant impact on the host immune system. Certain species from the *Chlorobi* phylum, like *Chlorobium*, are also recognized as sphingolipid producers. In IBD, sphingolipids are crucial in several key processes, such as maintaining mucosal barrier integrity, supporting receptor functions, and contributing to the production of sphingolipid-derived signaling molecules in epithelial and immune cells. Disruptions in sphingolipid metabolism have been implicated in the pathogenesis of IBD and other diseases, underscoring their importance in preserving gut health and regulating immune responses [66,67].

### 3.7. N-Acyl Amide

N-acyl amides (NAAs), including endocannabinoids, regulate diverse cellular functions partly due to their interaction with G-protein-coupled receptors (GPCRs). These receptors are likely key mediators of host-microbial interactions within the human microbiota. The microbiota produces NAAs that closely mimic the function of endogenously produced signaling molecules, particularly endocannabinoids. Specifically, the microbiota can generate six families of N-acyl amides: N-acyl glycine, N-acyloxyacyl lysine, N-acyloxyacyl glutamine, N-acyl lysine/ornithine, N-acyl alanine, and N-acyl serinol [68,69,70]. 

Endocannabinoids, integral to this biochemical dialogue, are involved in regulating several critical physiological functions, including those related to obesity, inflammation, and gut-barrier integrity. The endocannabinoid system, in particular, is pivotal in gut inflammatory diseases such as UC and CD. However, despite the well-established involvement of the gut microbiota, the endocannabinoid system, and intestinal inflammation in these conditions, a significant gap remains in the scientific literature regarding the precise interactions between these three components [71,72].

### 3.8. Vitamins

Changes in the gut microbiota composition affect the production of various vitamins in IBD patients, including vitamins B, E, and D. 

#### 3.8.1. Vitamin B

Vitamin B is a group of water-soluble nutrients having a crucial role as cofactors in various metabolic pathways. These vitamins are primarily absorbed in the small intestine, facilitated by specific transporters, while those synthesized by gut microbiota are absorbed in the colon, where microbial populations are densest. Since human organisms cannot produce vitamin B de novo, it must be obtained mainly through diet, with an additional contribution from gut bacteria [73]. Gut microbiota can influence the utilization of vitamin B by either producing or consuming it, which affects its availability to the host. The gut microbiota also impacts the absorption process of vitamin B, which is essential for intestinal and overall health by supporting various metabolic and immune functions [74].

Various vitamins B have been identified to be essential in maintaining gut homeostasis. One is vitamin B3 (niacin), which may play an important role. A study by Fangmann et al. observed that obese individuals with a low intake of niacin experienced reduced microbial diversity and a lower population of *Bacteroidetes* in their gut. To counter these effects and reduce the potential side effects of niacin, researchers developed delayed-release microcapsules that specifically target the ileocolonic region. These microcapsules, designed to deliver vitamin B3 directly to the gut microbiota, were found to significantly enhance the abundance of *Bacteroidetes* [75]. Other studies have reported that niacin influences the bacterial community, which impacts the production of beneficial SCFAs in the colon of piglets [76].

#### 3.8.2. Vitamin E

Studies indicate that vitamin E can influence the composition of the gut microbiota, altering its balance and promoting beneficial changes, such as increasing the number of butyrate-producing microorganisms, which have anti-inflammatory effects. Vitamin E, particularly in the form of γ-tocopherol, may improve microbiota composition in individuals with IBD and affect microbiota diversity. However, the effects vary depending on the dose and form of vitamin E, and the impact on the microbiota is not always consistent. Further research is needed to understand better vitamin E’s mechanisms and consistent effects on the gut microbiota [77,78,79].

#### 3.8.3. Vitamin D

Vitamin D enhances the intestinal barrier function, regulates inflammatory responses, and affects the gut microbiota by influencing microbial diversity and abundance, particularly by promoting beneficial bacteria and suppressing pathogenic ones. On the other hand, the microbiota affects vitamin D metabolism through microbial production of metabolites that can influence its activation and function. Any imbalance in the gut microbiota or vitamin D deficiency is associated with various health conditions, including autoimmune diseases, infections, and IBD [80]. Research in humans has identified notable links between vitamin D levels and the composition of the gut microbiota. In a cross-sectional study involving healthy individuals, higher vitamin D intake was found to be inversely related to the abundance of the *Prevotella* while showing a strong positive association with the presence of the *Bacteroides*, both belonging to the *Bacteroidetes* phylum [81].

### 3.9. Polyamines

Toll-like receptor 2 expression modulating intestinal epithelial integrity requires polyamines [82]. Commensal bacteria produce polyamines from L-arginine, which is a central intestinal metabolite [83]. Expression of this receptor is markedly elevated in the intestinal lining of patients with CD [84]. 

## 4. Interactions between Host Genes and Microbiota

**Figure 1:**
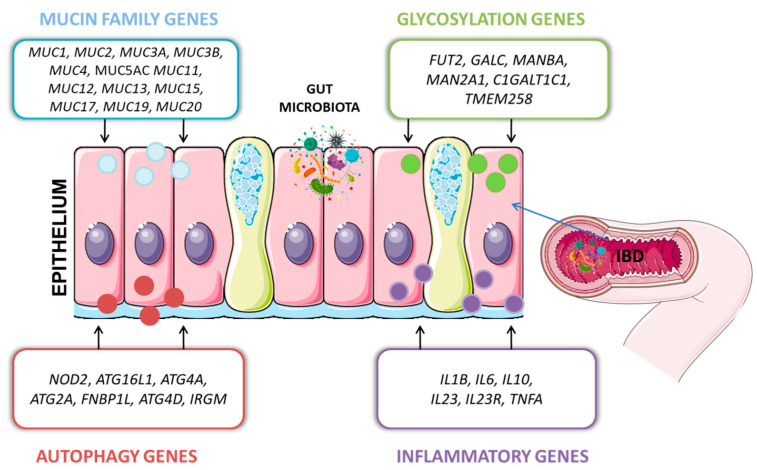
Genes having an impact on gut microbiota.

The impact of genetic factors in IBD conditioning has been known for over three decades. However, the interactions between host genetic variants and the gut microbiota constitute a separate issue. The drivers of IBD in the human genome that have been described include nearly 240 risk loci, many well-known and documented, such as *NOD2*, *ATG16L1*, *IGRM*, *CARD9*, and *IL23R* [85]. The basic genetic background of IBD was also described in our previous paper [86]. Currently, the importance of those host genes is highlighted in that they directly influence microbial colonization in the intestine [35] (Figure 1). The latest studies focus on this relationship (including glycosylation level, mucins, and autophagy gene expression level), considering this double-sided host-microbe impact as an important part of IBD pathogenesis.

### 4.1. Glycosylation Genes

The latest research provides evidence that intestinal epithelial glycosylation is an underappreciated process that is linked with all these risk factors of IBD. An association was found between IBD cases and increased expression of truncated O-glycans, as well as altered expression of terminal glycan structures. Epithelial glycans play an important role in regulating the gut microbiota because they provide bacterial ligands, nutrients, and proper colonization of commensal bacteria. IBD risk genes, glycosyltransferase mislocalization, disturbed glycosyltransferase, and glycosidase expression can cause changes in the glycome. What is important to note is that glycome changes are driven by dysbiosis. For example, *Salmonella* can directly alter human glycan biosynthesis [87]. Impairment of the glycosylation profile leads to disruption of the mucus layer, glycan–lectin interactions, host-microbe interactions, and mucosal immunity, ultimately contributing to IBD pathogenesis [5]. The primary genes involved in IBD pathogenesis implicated in glycosylation are *FUT2*, *GALC*, *MANBA*, *MAN2A1*, *C1GALT1C1*, and *TMEM258* [88] (Table 1).

The major glycosylation gene, *FUT2*, is expressed in the colon, duodenum, and small intestine. The gene synthesizes an enzyme (fucosyltransferase 2) responsible for attaching fucose sugar residues to proteins and lipids in the host mucosa. Epithelial fucosyltransferase 2 creates a symbiotic environment for the host and commensal bacteria. Reduced *FUT2* expression or the lack of a functional variant increases the risk of developing IBD [89]. In the group of mice with Fut2 deficiency, colitis, acute inflammation, large infiltrates, and mucosa ulceration were observed. The intestinal barrier was also disturbed in the animal model, which further exacerbated colon damage. The comparative analysis of the 16S rRNA gene of the microbiota of *Fut2+* and *Fut2*- individuals showed a reduced amount of beneficial bacteria from the *Ruminococcaceae* and *Muribaculaceae* family, while the pathogenic microorganisms such as *Bilophila*, *Escherichia*, *Enterorhabdus* were found to be increased in the *Fut2-* cohort [90].

Other studies in IBD patients showed that the *FUT2* variant was associated with a decreased abundance of *Escherichia*, which usually binds to fucosylated oligosaccharides. The *FUT2* gene also correlates with an increased quantity of *Alistipe* and *Phascolarctobacterium*, capable of inducing CD8 cells. The *FUT2* gene mutation participates in the pathogenesis of IBD by changing the microbial composition by means of reducing the number of binding sites of adjacent bacteria. Decreasing numbers of adhering bacteria may allow the overgrowth of bacteria that induces T-lymphocytes and the intestinal epithelium’s inflammatory process [91].

Another gene implicated in the glycosylation process is *MAN2A1*, encoding the alpha-mannosidase 2 enzyme, which is correlated with the pathogenesis of UC. Studies show that this gene is responsible for the inflammatory state in connection with the increased recruitment of neutrophils [92].

Furthermore, the *C1GALT1C* gene coding for galactosyltransferase also impacts microbial composition in male mice. Loss of chaperone 1β3GalT from intestinal epithelial cells (IEC) disrupts the mucus layer, leading to dysbiosis and sudden inflammation. Sequencing studies demonstrated an 11-fold reduction in *Bacteroides* and a three-fold increase in pathogenic *Helicobacter* microbes in subjects with IBD [93].

### 4.2. Autophagy Genes

Recent investigations have identified autophagy-related genes among genetic factors playing a role in IBD etiology [88] (Table 1). Autophagy is crucial for maintaining intestinal homeostasis and the proper immune response. This process was reported to regulate intestinal barrier function by inducing lysosomal degradation of the tight junction protein (claudin 2), thus decreasing epithelial permeability [94]. Dysfunctional autophagy leads to altered epithelial function, dysbiosis, and an aberrant immune response. The GWAS studies of variants identified a group of IBD susceptibility autophagy genes: *NOD2*, *ATG16L1*, *IGRM*, *CARD9*, and *IL23R* [95]. Molecular analysis made it possible to formulate a research hypothesis on the significance of the correlation between the genetic aspect of the host and intestinal microbes and to offer an answer to the following question: ‘What should be the optimal environment for the appropriate microbial level?’ The first gene identified was *NOD2* (*CARD15*) in the IBD1 (IBD susceptibility locus 1, OMIM#266600) region on chromosome 16q12.1. The gene is highly expressed in Paneth cells located in the intestinal crypts and partially in the intestinal epithelium [96]. Well-described gene polymorphisms of CD are the following variants: rs2066846 Leu1007fs; rs2066845, Gly908Arg; and rs2066844 Arg702Trp (Table 1). The C allele of rs2066845 carriers significantly influenced the change in the microbiota’s composition through the increased level of fecal bacteria *Erysipelotrichaceae* [97]. However, the dominant predictor of CD was the Leu1007fs variant, which causes a frameshift. In effect, it was associated with complications of the disease: 79% of homozygous carriers among patients required hospitalization and ileal surgery [98]. Functional loss from homozygotes leads to a limited regulation of the host’s response to the gut microbes and changes in the microflora. There was a decrease in *Clostridia* and an increase in *Proteobacteria* in CD patients. The risk variants were also correlated with altered levels of *Faecalibacterium* and *E. coli* [99]. However, similar conclusions in studies with mice showed a high cluster load of *Bacteroidetes* and *Firmicutes* in the distal part of the intestine among CD homozygotes. In addition, these studies demonstrated that *NOD2* plays an elementary role in the anti-microbial activity of Paneth cells and the proper regulation of the intestinal microflora. In addition, risk alleles predispose to an increase in the pathogenic taxa disrupting the intestinal microbiota—*Yersinia*, *Campylobacter*, *Citrobacter*, *E. coli*, *Helicobacter*, *Listeria*, *Mycobacteria*, *Pseudomonas*, and *Staphylococcus*, *Erysipelotrichaceae*. It is worth noting that *NOD2* interacts with other genes *IRGM* and *ATG16L1*, intensifying the process of autophagy. Altered *IRGM* expression affected the microflora of the healthy group by reducing the abundance of the butyrate-producing genus *Roseburia* [100]. Studies indicate that variant T300A in the *ATG16L1* gene leads to impaired autophagy and increased pro-inflammatory cytokine production. CD patients homozygous for variant T300A in the *ATG16L1* gene exhibit an increased abundance of pathobionts such as *E. coli, Bacteroidesfragilis*, and *Fusobacteriaceae*. The T300A variant of the *ATG16L1* gene is also correlated with a reduced amount of the commensal bacterium. These bacteria provide an optimal environment in the intestine and revitalize the host’s mucus layer, thus maintaining its integrity. T300A polymorphism contributes to a reduction in *Akermansia muciniphila* in IBD patients. In addition, the gene variant led to increased recruitment of *Muscipirillum schaedleri*, which may propagate colitis during UC [101]. Also, other genes involved in the autophagy process were identified as associated with CD pathogenesis, such as *ATG4A*, *ATG2A*, and *ATG4D*, coding for autophagy-related cysteine peptidases [88].

### 4.3. Mucins

Mucins, large glycoproteins decorated with O-linked glycans, are IBD host-risk factors directly influencing microbiota, which play a significant role in host-microbiome interactions through the ability to gel (moisturize), create chemical barriers, and participate in the cell signaling pathway [102]. HUGO Gene Nomenclature Committee has classified 21 mucin genes, of which 13 are expressed in the intestinal epithelium (Table 1) [103].

Some pathogens may downregulate mucin production to disrupt the mucus barrier integrity. In particular, *Helicobacter pylori* and *Clostridioides difficile* infections are associated with disrupted mucin synthesis and mucus barrier function. *H. pylori*-infected patients indicated a significant decrease in the gene *MUC5AC* expression level, coding for mucin 5AC. IBD patients exhibit an altered mucus barrier or mucin production. This is supported by some studies, in which animal models with disrupted barrier integrity manifested IBD-like syndromes. Decreased expression of *MUC5AC* was observed in patients with exacerbated enteritis, and extended molecular studies showed that endoscopic treatment of UC patients increased its expression. However, work is still ongoing to understand the functional role of MUC5AC in inducing inflammation in the pathogenesis of IBD [104]. In 2020, a patient group study showed that the expression of human *MUC5AC* is correlated with active UC [105]. However, 55% of healthy subjects in the control group did not express *MUC5AC*. These results showed that the mucin expression was restricted in healthy, non-inflammatory tissue. The same study was conducted in a mouse model, where a knock-out of *Muc5ac* was performed. Persistent loss of the functional variant enhanced the interaction of the bacteria with the epithelial host. Sustained contact between *Helicobacter pylori* and the host intestinal mucosa led to an increase in the level of neutrophilia and then initiated increased production of inflammatory cytokines: interleukin-10 (IL10) and C-X-C motif chemokine ligand 16 (CXCL16) [106]. In conclusion, the induction of human *MUC5AC* expression can directly strengthen the protective barrier by maintaining the integrity and thickness of the mucin layer, which plays a preventive role in inflammation associated with UC [107]. Studies also identified the *MUC19* gene as a risk locus for IBD [108]. 

On the other hand, the link between UC and the mucin layer is well established. UC patients display a thinner mucus layer due to impaired MUC2 production and secretion. The *MUC2* gene induces the production of the MUC2 protein in the gut, which is the major component of the mucin gel layer [109]. Animal model studies also confirmed that the deletion of *Muc2* conditioned the reduced thickness of the protective mucin layer and led to the development of colonic inflammation in patients with UC [110]. Current evidence suggests that rare *MUC3A* alleles can encode mucin proteins with specific conformational changes. Altering the protein’s conformation can result in reduced glycosylation because the processes of O-glycosylation and N-glycosylation depend on the protein’s structure. Less glycosylation correlates with increased sensitivity to intestinal bacterial proteases and mucin degradation, thereby breaking the continuity of the barrier [111]. *MUC9* and *MUC20* gene expression was significantly decreased in patients with active UC compared to both the remission group and controls [112].

### 4.4. Cytokines

It is also known that impaired protective function of the intestinal barrier promotes the synthesis of cytokines. Thus, the complex pathogenesis of IBD includes genes encoding proteins involved in the immune response, such as *IL1B*, *IL6*, *IL10*, *IL23*, *IL23R*, and *TNFA* [113] (Table 1). The latest research from 2022 shows that the decreased level of IL6 contributed to the dysbiosis of the intestinal microflora in mice, increasing the abundance of Gram-negative bacteria with impaired metabolic pathways. Decreased *IL10* expression was associated with a greater abundance of *Bacteroides*, *Prevotella*, and *Rikenella* [114]. Accordingly, an increased serum concentration of interleukin-23 receptor (IL23R) was noted in IBD patients. The disturbed balance of microorganisms leads to the growth and colonization of pathogens, which promotes an increased immune response and the development of IBD. *Klebsiella pneumoniae* invade intestinal epithelial cells and interact with macrophages to cause the secretion of IL1B and TNFα [115]. Additionally, the disturbing expression of genes responsible for autophagy, which leads to abnormal colonization of AIEC pathobionts, has been described as causing enhanced inflammation and the synthesis of pro-inflammatory cytokine TNFα [116]. We also observed that the species *Dorea* negatively correlates with the interferon-gamma (IFNG) response. Moreover, the species mentioned (*Streptococcus parasanguinis* and *Streptococcus australis*) were associated with IFNγ production. Other species, such as *Streptococcus mitis*, *Streptococcus oralis*, and *Streptococcus pneumoniae*, were also associated with *IL1B* overexpression. On the other hand, *Bifidobacterium adolescentis* was inversely related to the production of TNFα, which highlights the potential cytokine and species specificity in the group of IBD patients [117].

**Table 1:** Host genetic variants and their impact on gut microbiota in IBD.

Crohn’s Disease (CD)				
Gene, Full Name	Genetic Variant rs Number, Minor Allel, Consequence	Location	MAF (%)	Function/ Impact on the Host Microbiota
***ATG16L1*** Autophagy-related 16 like 1	rs12994997 (A) intron variant	2q14.1	39	**↑** The risk allele (A) increases abundance of pathogenic symbionts in the intestinal mucosa: *Enterobacteriaceae*, *Bacteroidaceae*, and *Fusobacteriaceae*. **↑** The protective allele (G) increases the number of commensal bacteria *Lachnospiraceae* [118].
	rs10210302 (T) 2kb upstream variant	2q14.2	39	Variant C/C is significantly associated with the protection of IBD patients in the Indian population OR = 0.89 (0.71–1.13) [119].
	rs2241880 (G) missense variant p.Thr216Ala	2q14.1	40	A significant difference in the incidence of *Listeria monocytogenes* and *Yersinia enterocolitica* pathobionts in patients with CD compared to the control group (*p* < 0.05); **↑** The variant T300A leads to impaired autophagy and increased pro-inflammatory cytokine production; **↑** Patients homozygous for variant T300A in the *ATG16L*1 gene exhibit an increased abundance of pathobionts such as *E. coli*, *Bacteroidesfragilis*, and *Fusobacteriaceae*. *Muscipirillum schaedleri*; ↓ Reduction of *Bacteroidetes* and *Firmicutes* [120,121].
	rs6754677 (A) intron variant	2q14.2	37	The homozygous genotype A/A showed a risk of developing CD, is associated with terminal ileitis as well, and is related to autophagy [122].
***NOD2*** nucleotide-binding oligomerization domain containing 2	rs2066844 missense variant p.Arg702Trp	16q11.2	1	↑ Increased level of *Enterobasscteriaceae* family and *Helicobacter pylori*—a risk factor for colon cancer in CD patients [97].
	rs2066845 missense variant p.Gly908Arg p.Gly908Cys	16q11.2	1	↓ Reduction of *Bacteroidetes* and *Firmicutes*; ↑ Additionally, the C allele was significantly associated with an increase in relative abundance in the fecal bacterial family *Erysipelotrichaceae* [97,99].
	rs2066847 (CC/CCCC) frameshift variant p.Leu1007Pro(fs)	16q12.1	1	Impaired synthesis of pro-inflammatory cytokines (IL1B) and dendritic cells, leading to deregulation of the host’s antimicrobial defense; ↑ Increased abundance of pathobionts and permeability gut [98].
	-	-	-	↑ Increased pathogenic taxa: *Yersinia*, *Campylobacter*, *Citrobacter*, *E. coli*, *Helicobacter*, *Listeria*, *Mycobacteria*, *Pseudomonas*, and *Staphylococcus* [123].
***IRGM*** immunity-related GTPase M	rs11741861 (G) intron variant	5q14.3	16	↓ The risk variant reduces the abundance of anaerobic bacteria, butyrate-producing *Roseburia* in patients with IBD. The protective barrier mucosa of the colon is compromised and, as a result, inflammation is triggered [100].
	rs13361189 (C) intergenic variant	5q14.3	30	Alteration in the intensity of inflammation of the intestinal mucosa as a result of the implication of an accelerated immune response [124].
	rs10065172 (T) missense variant p.Leu105=	5q14.3	30	↑ Increased susceptibility to CD in individuals of European patients (*p* = 0.008); Haplotype T/T influenced the binding site of a specific microRNA, causing the deregulation of *IRGM*-dependent xenophagy bacteria in patients with CD; ↑ In addition, the T/T genotype is also associated with an increased level of expression of the cytokine TNFα in the peripheral blood, influencing inflammation [95,124].
**Ulcerative colitis (UC)**				
***MUC13*** mucin 13	rs1127233 (G) missense variant p.Arg503Ser	3q21.2	23	Variant correlated with UC *p* = 0.0003; disturbed *MUC13* gene expression correlated with the NFkB pathway can lead to a loss of membrane integrity and, thus, permeability [125].
**Inflammatory Bowel Disease (IBD)**				
***MUC1*** mucin 1	-	-	-	*MUC1* codes for the main mucus component, which is the physical barrier that protects the intestinal epithelium from intestinal bacteria. MUC1 overexpression and hypoglycosylation have been reported in Muc1-knockout IBD mice showing increased damage to the small intestine following infection with *C. jejuni* [126].
	rs4072037 (C) synonymous variant p.Thr22=	1q22	37	*↑* Increase in the abundance of *Ochrobactrum* [127].
***MUC2*** mucin 2	rs2856111 (T) missense variant Leu58Pro	11p15.5	27	Reduced gene expression is associated with a thinner mucus layer in UC patients—particularly at the site of inflammation due to the reduction of goblet cells [128].
	rs11825977(A) missense variant p.Val116Met	11p15.5	12	Decreased MUC2 mRNA expression increases the risk of inflammation and intestinal dysbiosis [125].
***MUC3A*** mucin 3A	-	7q22.1	-	Rare alleles change the conformation of the proteins produced. The conformation affects the glycosylation process, which increases the sensitivity to bacterial proteases, and, thus, breaks the continuity of the protective gel barrier [111].
***MUC5AC*** mucin 5AC	rs35783651 (G) missense variant p.Ser221Arg	11p15.5	10	Protective role by participating in the healing of mucosal epithelial wounds and regulating MGL; *H. pylori*-infected patients indicated a significant decrease in *MUC5AC* expression level [106].
***MUC19*** mucin 19	rs11564245 (C) missense variant p.Asp803His	12q12	5	Increased susceptibility to CD in the group of patients [3,108].
	rs4768261 (T) missense variant p.Ser1226Phe	12q12	5	
***CARD9*** caspase recruitment domain family member 9	rs4077515 (T) missense variant p.Ser12Asn/Ile	9q21.3	37	Innate immune response to peptidoglycan, a macromolecule in the bacterial cell wall; Aberrant activation of NF-κB and inflammatory factors in response to *Aspergillus fumigates*, contributing to intestinal inflammation [129].
	rs10781499 (A) synonymous variant p.Pro42=	9q34.3	37	↓ Decrease butyrate acetate converting bacteria—*Roseburia* spp. [100].
	rs10870077 (G) intron variant	9q34.3	37	↑ Increased risk of UC development by modulating the signaling pathway affecting the inflammatory response [130].
***FUT2*** fucosyltransferase 2	*Fut2-*	19q13.33	-	↓ Reduced beneficial bacteria from *Ruminococcaceae* and *Muribaculaceae*, while the pathogenic microorganisms, such as ↑ *Bilophila, Escherichia, Enterorhabdus, Alistipe*, and *Phascolarctobacterium*, were increased in the cohort [90,91].
***C1GALT1C*** core 1 synthase, glycoprotein-N-acetylgalactosamine 3-beta-galactosyltransferase 1	*-*	7p22.1-p21.3	-	Studies demonstrated an 11-fold reduction of *Bacteroides* and a 3-fold increase of pathogenic *Helicobacter* microbes in the IBD cohort [93].
***IL1B*** interleukin 1 beta	-	2q14.1	-	Increased ILIB level by attack and colonization of *Klebsiella pneumoniae*, *Streptococcus mitis*, *Streptococcus oralis*, and *Streptococcus pneumoniae* [115].
***IL6*** interleukin 6	-	1q21.3		The deficiency of IL6 contributes to the dysbiosis of the gut microbiota and increases the abundance of Gram-negative bacteria [131].
***IL10*** interleukin 10	rs1800896 (A) 2kb upstream variant	1q32.1	27	↓ Loss of IL10 receptor function—induction of inflammation in severe course of UC; Allele A was associated with UC *p* = 0.011 in in Mexican cohort; ↑ Increased susceptibility to fungal infections with *Candida albicans*; ↓ Decreased *IL10* expression is associated with ↑ increased *Bacteroides*, *Prevotella*, and *Rikenella* [114].
***IL23R*** interleukin 23	rs1004819 (A) intron variant	1p.31.3	40	Early age of onset of the disease in the Polish population [132].
	rs76418789 (A) missense variant p.Gly149Arg	1p.31.3	1	SNP associated with IBD in the Korean population (*p* = 0.0096) [133].
	rs11209026 (A) missense variant p.Arg381Gln	1p.31.3	2	Protective effect in CD but related to UC; ↑ Increased abundance of *Christensenellaceae*, *Bacteroides caccae*, and a ↓ decrease in the commensal bacteria *Faecalibacterium prausnitzii* [113]
	rs2201841 (G) intron variant	1q11	40	Significant association between polymorphisms and UC, especially in Caucasiasns [134].
***IFNG*** interferon gamma	-	12q15	-	↑ Increased level of taxa: *Dorea*, *Streptococcus parasanguinis* and *Streptococcus australis* [117].

MAF—minor allele frequency; ↑ mean increased; ↓ mean decreased.

## 5. Microbial-Based Therapies

Among global research on IBD pathogenesis, there is a visible trend to move from microbial profiling into a study of whole genomic sequences of microbes and into host-microbe interactions, which should provide the development of microbiota-based diagnostics and targeted therapy for IBD. Recent studies in this field attempted an integrative analysis of host, microbial, and multi-omics risk factors to find an algorithm for precisely characterizing CD and UC profiles. Understanding the role and pathways of microbial biomarkers in the assessment of disease activity and treatment outcomes is critical to the monitoring and treatment of IBD. The knowledge from this integrative multi-omics analysis is necessary to develop targeting microbiota-based therapeutic approaches, which is a promising strategy to alleviate and cure this inflammatory disease.

One therapeutic option for IBD patients is fecal microbiota transplantation (FMT) from healthy individuals with rich microbial composition. FMT allows for the transfer of a part of the entire ecosystem, not just the microbes. It helps in calming inflammation in patients and is more effective than oral intake of probiotics, but it is controversial. FMT can be used as therapy in IBD in case the antibiotics are ineffective. Particularly promising probiotic bacteria are *Faecalibacterium prausnitzii*, *Akkermansia muciniphila*, and some *Roseburia* strains.

In recent years, phage-based therapy has focused new attention on IBD. It was proven in several clinical trials in adults and children as a safe therapeutic option without adverse events. Moreover, phages constitute a majority of viruses in the human gut. Phages could target potentially disease-causative bacteria, such as *Ruminococcus gnavus*, *E. coli*, *Bacteroides fragilis*, or fungi, such as *C. albicans*, enriched in the human IBD intestine.

We are hopeful to observe the progress of scientific research in this area and believe that, shortly, IBD will be a curable disease.

## 6. Conclusions

**Figure 2:**
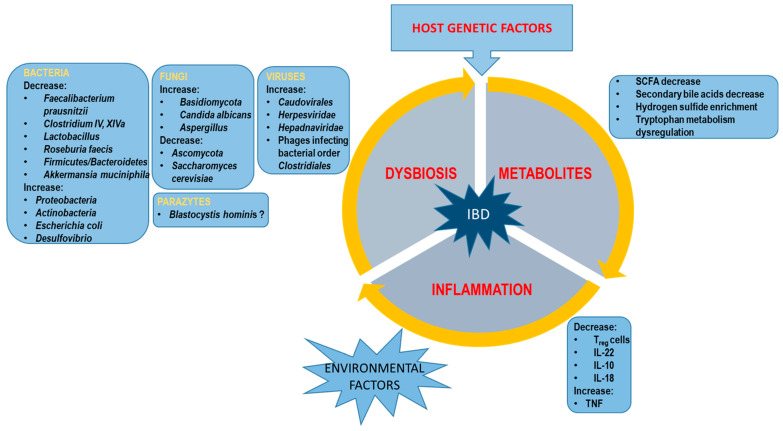
Scheme of factors implemented in IBD pathogenesis.

In the last 10 years, there has been an intensive development of research on the intestinal microbiota and the metabolome, which has allowed for a broader understanding of the complexity of IBD pathogenesis (Figure 2). However, it is still not entirely clear. NGS technology opens up a new area in microbiota identification, allowing scientists to analyze and compare whole microbial communities, including their composition, interactions, and functions. Despite the often-inconsistent results of global research, it has been possible to identify the “pathogenic” and “protective” microbiota in the context of IBD development. Similarly, in terms of metabolites, examples of those that intensify inflammation and those that help calm it down have been identified (Figure 2).

The weakness of research conducted around the world aimed at characterizing the microbiota associated with IBD is certainly the diverse methodology used to identify the microbiota—namely, analysis of different regions (V1–V9) of the 16S rRNA gene and the diversity of NGS platforms, which affects the final qualitative and quantitative result of identified taxa. Similarly, for methods to identify metabolites associated with IBD, targeted and untargeted compound identification approaches are used. Notably, untargeted deep analytical approaches give us the potential to discover new important small molecules.

## Data Availability

Manuscript based on the public available data in PubMed.
